# Association of body mass index and waist circumference with type 2 diabetes mellitus in older adults: a cross-sectional study

**DOI:** 10.1186/s12877-022-03145-w

**Published:** 2022-06-07

**Authors:** Kaizhi Bai, Xuejiao Chen, Rui Song, Wenlong Shi, Songhe Shi

**Affiliations:** College of Public Health, Zhengzhou University, Zhengzhou, Henan China

**Keywords:** Type 2 diabetes mellitus, Body mass index, Waist circumference, Anthropometric indicators, Older adults

## Abstract

**Background:**

The prevalence of obesity and diabetes is rising. The aim of this study was to determine the association of body mass index (BMI) and waist circumference (WC) with type 2 diabetes mellitus (T2DM) in the elderly and to compare the discriminatory abilities of BMI, WC and other anthropometric indicators, including waist-to-height ratio (WHtR), body adiposity estimator (BAE) and body roundness index (BRI) for T2DM.

**Methods:**

This cross-sectional study included 69,388 subjects aged ≥ 60 years living in Xinzheng, Henan Province, from January to December 2020. The data came from the residents’ electronic health records of the Xinzheng Hospital Information System. Logistic regression was used to examine the relationships. Fully adjusted models adjusted for age, sex, place of residence, alcohol consumption, smoking, physical exercise, SBP and RHR. The area under the receiver operating characteristic curve (AUC) was used to compare the discriminatory ability of different anthropometric indicators for T2DM under the influence of potential risk factors.

**Results:**

After adjusting for multiple covariates, compared with the first BMI quintile, the odds ratios (ORs) and 95% confidence intervals (CIs) from the second to fifth quintile for T2DM were 1.416 (1.335–1.502), 1.664 (1.570–1.764), 1.879 (1.774–1.990) and 2.156 (2.037–2.283), respectively. Compared with the first WC quintile, the ORs and 95% CIs from the second to fifth quintiles for T2DM were 1.322 (1.244–1.404), 1.549 (1.459–1.643), 1.705 (1.609–1.807) and 2.169 (2.048–2.297), respectively. Among men, compared with other anthropometric indicators (BMI, WHtR, BAE and BRI), WC showed the highest AUC (AUC: 0.629; 95% CI: 0.622–0.636). Among women, the AUCs of BMI (AUC: 0.600; 95% CI: 0.594–0.606), WC (AUC: 0.600; 95% CI: 0.593–0.606) and BAE (AUC: 0.600; 95% CI: 0.594–0.607) were similar, and the AUCs of BMI, WC and BAE were higher than WHtR, BRI.

**Conclusions:**

All anthropometric indicators were positively associated with T2DM. In men, WC with the strongest positive association with T2DM was the best predictor of T2DM. In women, BMI was most strongly associated with T2DM, and the predictive powers of BMI, WC and BAE were similar. After adjusting the potential confounding factors including age, sex, place of residence, alcohol consumption, smoking, physical exercise, SBP and RHR, the effect of these factors was eliminated, the findings were independent of the covariates considered.

**Supplementary Information:**

The online version contains supplementary material available at 10.1186/s12877-022-03145-w.

## Background

In recent years, diabetes has been a substantial public health burden worldwide. The global prevalence of diabetes has reached 10.5% (536.6 million people) and will reach 12.2% (783.2 million people) by 2045. Middle-income countries had the largest increase in the prevalence of diabetes. The economic cost was $966 billion in 2021 and will be $1,054 billion in 2045 [1]. The prevalence of diabetes in Chinese adults was 12.8% (ADA criteria including the addition of HbA1c) in 2017. The total number of diabetes patients in mainland China was approximately 129.8 million (70.4 million men and 59.4 million men). The prevalence of diabetes was 28.8% in individuals 60–69 years old and 31.8% in individuals over 70 years old [2].

Diabetes can cause many complications: macrovascular complications, including coronary heart disease, stroke and peripheral vascular disease, and microvascular complications, such as end-stage renal disease, retinopathy and neuropathy, and lower-extremity amputations [3]. The risks of all-cause [4] and cardiovascular disease (CVD) mortality [5] were significantly increased in patients with diabetes. Meanwhile, the quality of life of people with diabetes may decrease [6].

Diabetes has multiple risk factors, such as overweight, obesity, unhealthy diet, poor lifestyle habits, and increased age [7–10]. In recent years, the proportion of people with obesity (BMI ≥ 30 kg/m^2^) has been rising substantially worldwide [11]. The prevalence of overweight (25 kg/m^2^ ≤ BMI < 30 kg/m^2^) and obesity (BMI ≥ 30 kg/m^2^) among Chinese adults was 28.1% and 5.2%, respectively [12]. The prevalence of central obesity [waist circumference (WC) ≥ 90 in males and ≥ 85 in females] in Chinese adults was 29.1% (28.6% in males and 29.6% in females), and the estimated total number was 277.8 million (140.1 million males and 137.7 females) [13].

Some articles have studied the relationship between obesity and type 2 diabetes mellitus (T2DM). However, the relationship between obesity and T2DM has always been estimated by grouping body mass index (BMI) or waist circumference (WC), so it is difficult to obtain the precise dose–response relationship. Few studies have examined the interaction between BMI and WC for T2DM. Therefore, this research studied the relationship between BMI and WC and T2DM in elderly individuals, including the dose–response relationship based on restricted cubic spline plots and the additive interaction between WC and BMI for T2DM. The receiver operating characteristic curve (ROC) was used to compare the predictive ability of WC, BMI and other anthropometric indicators for T2DM, including WHtR, BAE and BRI. These indicators have recently been used to study cardiovascular disease, hypertension, mortality and so on [14, 15], so we calculated and analyzed these indicators and compared these indicators with BMI and WC.

## Method

### Population

The subjects of this cross-sectional study were adults aged ≥ 60 years in Xinzheng, Henan Province, Central China. The data came from the residents' electronic health records in the Xinzheng Hospital Information System from January to December 2020. Doctors set up health records for each resident at their first hospital visit or health examination, and participants aged 60 or older could receive free health examinations. At the start of the study, 70,161 elderly adults were eligible for the study. We excluded participants with the following conditions: (1) missing information for marital status, drinking, smoking, exercise, resting heart rate (RHR), systolic blood pressure (SBP), diastolic blood pressure (DBP), WC or BMI (*n* = 532); (2) the values of the variables above were illogical (*n* = 241). Ultimately, the study included 69,388 participants. The data screening flow chart is presented in Supplementary Fig. 1. Informed consent was obtained from the subjects, and this study was approved by the Ethics Committee of Zhengzhou University (Reference Number: ZZUIRB2019-019).

### Data collection

Demographic and clinical information was collected at the health checkup for participants. Demographic information included sex (male/female), age, place of residence (urban/rural), marital status, alcohol consumption, smoking, and physical exercise. Smoking included never smokers, former smokers and current smokers. Alcohol consumption and physical exercise were divided into four categories: never, once in a while, more than once a week and every day. Clinical data included anthropometric measurements, laboratory investigations, and self-reported disease history. Participants wearing light clothing took off their shoes, and then their weight and height were measured. BMI was calculated as weight in kilograms divided by the square of height in meters. WC was measured at the midpoint of the distance between the lowest costal ridge and the upper border of the iliac crest.

Other anthropometric indicators included WHtR, BAE and BRI.

WHtR was calculated by dividing WC by height, BAE = -44.988 + (0.503 × age) + (10.689 × sex) + (3.172 × BMI)-(0.026 × (BMI)^2^) + (0.181 × BMI × sex)-(0.02 × BMI × age)-(0.005 × (BMI)^2^ × sex) + (0.00021 × (BMI)^2^ × age), male = 0 and female = 1, and age is in years [16]. BRI = 364.2–365.5 × {1-[(WC/2π)^2^/(0.5height)^2^]}^1/2^ [17].

After participants fasted for 8 h, blood samples were collected to measure blood lipids and blood sugar. After participants had remained sitting for at least five minutes at rest, the SBP, DBP and radial pulse rate of the participants were measured twice by an electronic sphygmomanometer (Omron HEM-7125, Kyoto, Japan), and the mean value was recorded as the final result.

### Definition of T2DM

T2DM was defined as having a self-reported T2DM history, using insulin or oral hypoglycemic agents, or having FPG ≥ 7.0 mmol/L [18].

### Statistical analysis

Continuous variables were described as the means and standard deviations (SDs). Categorical variables are presented as numbers and proportions. The chi-square test or the Kruskal–Wallis test for categorical variables and ANOVA for continuous variables was used to compare the difference between quintiles of BMI or WC. The associations of BMI and WC with T2DM were analyzed in sex-specific quintiles by a logistic regression model, and ORs with 95% CIs of BMI and WC in categories and continuous variables were expressed in separate models. Model 1 was unadjusted. Model 2 adjusted for age and sex. Model 3 adjusted for the potential confounders, including age, sex, place of residence, alcohol consumption, smoking, physical exercise, SBP, RHR, because these potential confounders might affect the true relationships between the corresponding indicators and diabetes. Some studies have suggested a possible link between alcohol consumption and obesity and diabetes [19, 20], so we adjusted for that in our analysis. The dose–response association and the potentially nonlinear relationship of continuous BMI and WC with T2DM were explored by restricted cubic spline models with four knots. The stratified analysis was performed by sex subgroup using a logistic regression model to test the consistency of these relationships. We also performed additive interaction analysis between BMI and WC for T2DM with BMI and WC analyzed in two categories (BMI: BMI < 25 kg/m^2^ and BMI ≥ 25 kg/m^2^ [21]; WC: WC < 85 cm in females and < 90 cm in males, WC ≥ 85 cm in females and ≥ 90 in males [22]). We evaluated the existence of additive interactions by calculating the relative excess risk due to interaction (RERI), attributable proportion due to interaction (AP) and synergy index (S). RERI > 0, AP > 0 or S > 1 was considered a statistically significant additive interaction. Finally, the receiver operating characteristic (ROC) curve and related area under the ROC curve (AUC) were used to compare the capability of BMI, WC and other anthropometric indicators, including WHtR, BAE and BRI, to diagnosis T2DM, and the logistic regression model was used to estimate the related ORs and 95% CIs of WHtR, BAE and BRI for T2DM after adjusting for age, sex, place of residence, alcohol consumption, smoking, physical exercise, SBP, RHR. The Cohen’s d was utilized to estimate the effect size of anthropometric indicators [23]. Statistical analyses were performed using SPSS V 21 and R V 4.0.3. *P* < 0.05 with two-sided tests was considered statistically significant.

## Results

A total of 69,388 participants were enrolled, including 37,479 women and 31,909 men. The mean (SD) age was 71.4 (6.9) years. A total of 18,756 participants had T2DM, and the incidence rate was 27.0%. The baseline characteristics of the participants according to different BMIs and WC quintiles are shown in Table 1 and Supplementary Table 1. As BMI increases, the incidence of T2DM increases, SBP and DBP increase, the proportion of people who never exercise or drink decreases, and the proportion of current smokers decreases. Baseline characteristics grouped by WC showed a similar trend. The correlations among all the indices were showed in Supplementary Table 2. The unadjusted effect size of anthropometric indices between participants with or without T2DM was showed in Supplementary Table 3. The effect size of BMI was biggest among these anthropometric indices (Cohen’s d = 0.29, 95%CI: 0.28–0.31), and BRI was smallest (Cohen’s d = 0.24, 95%CI: 0.23–0.26).

**Table 1:** Baseline characteristics of the included participants according to different levels of BMI

**Characteristics**	**BMI, kg/m**.^**2**^					***P* Value**
	**First quintile**	**Second quintile**	**Third quintile**	**Fourth quintile**	**Fifth quintile**	
Male	BMI < 22.04	22.04 ≤ BMI < 23.75	23.75 ≤ BMI < 25.38	25.38 ≤ BMI < 27.36	BMI ≥ 27.36	
Female	BMI < 22.22	22.22 ≤ BMI < 24.06	24.06 ≤ BMI < 25.89	25.89 ≤ BMI < 28.13	BMI ≥ 28.13	
Number of participants	13,843	13,886	13,905	13,908	13,846	
Diabetes, %	2501 (18.1)	3393 (24.4)	3881 (27.9)	4271 (30.7)	4710 (34.0)	< 0.001
Age, years	73.3 ± 7.5	71.6 ± 7.2	71.0 ± 6.7	70.6 ± 6.4	70.4 ± 6.2	< 0.001
Women, %	7463 (53.9)	7503 (54.0)	7522 (54.1)	7527 (54.1)	7464 (53.9)	0.994
BMI, kg/m.^2^	20.4 ± 1.4	23.1 ± 0.5	24.8 ± 0.5	26.6 ± 0.7	30.1 ± 2.3	< 0.001
WC, cm	78.0 ± 6.5	82.8 ± 6.0	86.3 ± 6.1	89.7 ± 6.4	96.2 ± 8.3	< 0.001
BRI	3.32 ± 0.84	3.83 ± 0.82	4.28 ± 0.88	4.75 ± 0.95	5.73 ± 1.34	< 0.001
BAE	28.84 ± 6.62	31.95 ± 6.38	33.85 ± 6.40	35.94 ± 6.40	39.52 ± 6.75	< 0.001
WHtR	0.50 ± 0.04	0.52 ± 0.04	0.55 ± 0.04	0.57 ± 0.04	0.61 ± 0.06	< 0.001
Smoking, %						< 0.001
Never smokers	11,974 (86.5)	12,351 (88.9)	12,408 (89.2)	12,495 (89.8)	12,510 (90.4)	
Former smokers	211 (1.5)	208 (1.5)	225 (1.6)	220 (1.6)	219 (1.6)	
Current smokers	1658 (12.0)	1327 (9.6)	1272 (9.1)	1193 (8.6)	1117 (8.1)	
Alcohol consumption, %						< 0.001
Never	13,152 (95.0)	13,208 (95.1)	13,202 (94.9)	13,137 (94.5)	13,007 (93.9)	
Once in a while	378 (2.7)	406 (2.9)	413 (3.0)	445 (3.2)	492 (3.6)	
More than once a week	86 (0.6)	94 (0.7)	95 (0.7)	122 (0.9)	122 (0.9)	
Every day	227 (1.6)	178 (1.3)	195 (1.4)	204 (1.5)	225 (1.6)	
Physical exercise, %						< 0.001
Never	9690 (70.0)	9272 (66.8)	8745 (62.9)	8579 (61.7)	8680 (52.7)	
Once in a while	284 (2.1)	329 (2.4)	340 (2.4)	382 (2.7)	395 (2.9)	
More than once a week	628 (4.5)	809 (5.8)	968 (7.0)	1008 (7.2)	1006 (7.3)	
Every day	3241 (23.4)	3476 (25.0)	3852 (27.7)	3939 (28.3)	3765 (27.2)	
Rural areas, %	1550 (11.2)	2203 (15.9)	2479 (17.8)	2600 (18.7)	2544 (18.4)	< 0.001
RHR, beat	74.0 ± 11.8	73.3 ± 10.5	73.0 ± 10.7	73.2 ± 10.6	73.7 ± 10.8	< 0.001
SBP, mmHg	138.5 ± 20.0	139.3 ± 18.2	141.3 ± 18.4	142.2 ± 18.6	144.5 ± 18.7	< 0.001
DBP, mmHg	80.8 ± 10.8	82.4 ± 9.8	83.3 ± 9.9	84.1 ± 10.1	85.4 ± 10.4	< 0.001

Abbreviations: *BMI* body mass index; *WC* waist circumference; *SBP* systolic blood pressure; *DBP* diastolic blood pressure; *SD* standard deviation; *RHR* resting heart rate

The associations of BMI and WC with T2DM are presented in Table 2. After adjusting for other covariates including age, sex, place of residence, alcohol consumption, smoking, physical exercise, SBP and RHR, in Model 3, the OR (95% CI) per SD increase in BMI was 1.287 (1.265–1.309), and compared with the first BMI quintile, the ORs (95% CIs) of the second to fifth BMI quintiles for T2DM were 1.416 (1.335–1.502), 1.664 (1.570–1.764), 1.879 (1.774–1.990) and 2.156 (2.037–2.283), respectively. The OR (95% CI) per SD increase in WC was 1.299 (1.277–1.322) in Model 3, and compared with the first WC quintile, the ORs (95% CIs) of the second to fifth WC quintiles for T2DM were 1.322 (1.244–1.404), 1.549 (1.459–1.643), 1.705 (1.609–1.807) and 2.169 (2.048–2.297), respectively.

**Table 2:** Association between BMI, WC and T2DM

Variables	No. of cases	Model 1 OR (95% CI)	Model 2 OR (95% CI)	Model 3 OR (95% CI)
BMI (kg/m.^2^)				
Category				
Q1	2501	Reference	Reference	Reference
Q2	3393	1.466 (1.384, 1.554)	1.436 (1.354, 1.522)	1.416 (1.335, 1.502)
Q3	3881	1.756 (1.659, 1.859)	1.707 (1.612, 1.808)	1.664 (1.570, 1.764)
Q4	4271	2.010 (1.900, 2.126)	1.946 (1.838, 2.059)	1.879 (1.774, 1.990)
Q5	4710	2.338 (2.211, 2.472)	2.261 (2.137, 2.392)	2.156 (2.037, 2.283)
P for trend		< 0.001	< 0.001	< 0.001
Continuous (per SD)	18,756	1.333 (1.311, 1.356)	1.306 (1.284, 1.329)	1.287 (1.265, 1.309)
WC (cm)				
Category				
Q1	2364	Reference	Reference	Reference
Q2	3255	1.296 (1.221, 1.376)	1.326 (1.249, 1.408)	1.322 (1.244, 1.404)
Q3	3706	1.573 (1.484, 1.668)	1.577 (1.487, 1.673)	1.549 (1.459, 1.643)
Q4	4307	1.745 (1.648, 1.847)	1.752 (1.654, 1.855)	1.705 (1.609, 1.807)
Q5	5124	2.236 (2.114, 2.365)	2.251 (2.127, 2.381)	2.169 (2.048, 2.297)
P for trend		< 0.001	< 0.001	< 0.001
Continuous (per SD)	18,756	1.299 (1.277, 1.321)	1.316 (1.294, 1.339)	1.299 (1.277, 1.322)

Abbreviations: *OR* odd ratio; *CI* confidential interval; *BMI* body mass index; *WC* waist circumference; *SBP* systolic blood pressure; *RHR* resting heart rate

Model 1: unadjusted

Model 2: adjusted for age and sex

Model 3: adjusted for age, sex, alcohol consumption, place of residence, smoking, physical exercise, SBP, RHR

A stratified analysis was performed by subgroups of sex in Table 3, and the results showed that a higher BMI or WC was associated with a higher risk of T2DM in both male and female.

**Table 3:** Association between BMI, WC and T2DM by different sex

Variables	No. of cases	Model 1 OR (95% CI)	Model 2 OR (95% CI)	Model 3 OR (95% CI)
BMI (kg/m.^2^)				
Male				
Q1	997	Reference	Reference	Reference
Q2	1338	1.432 (1.308, 1.568)	1.412 (1.289, 1.546)	1.370 (1.250, 1.501)
Q3	1561	1.748 (1.600, 1.910)	1.713 (1.567, 1.872)	1.631 (1.491, 1.785)
Q4	1771	2.074 (1.901, 2.263)	2.029 (1.859, 2.214)	1.893 (1.732, 2.069)
Q5	2023	2.506 (2.300, 2.730)	2.443 (2.241, 2.663)	2.255 (2.064, 2.463)
P for trend		< 0.001	< 0.001	< 0.001
Continuous (per SD)	7690	1.359 (1.324, 1.394)	1.348 (1.314, 1.384)	1.344 (1.306, 1.383)
Female				
Q1	1504	Reference	Reference	Reference
Q2	2055	1.495 (1.385, 1.612)	1.452 (1.346, 1.568)	1.450 (1.343, 1.566)
Q3	2320	1.767 (1.640, 1.904)	1.703 (1.579, 1.836)	1.686 (1.563, 1.819)
Q4	2500	1.970 (1.830, 2.122)	1.885 (1.749, 2.031)	1.859 (1.724, 2.005)
Q5	2687	2.229 (2.070, 2.399)	2.130 (1.977, 2.294)	2.072 (1.922, 2.234)
P for trend		< 0.001	< 0.001	< 0.001
Continuous (per SD)	11,066	1.298 (1.270, 1.327)	1.279 (1.251, 1.309)	1.253 (1.226, 1.280)
WC (cm)				
Male				
Q1	742	Reference	Reference	Reference
Q2	1426	1.405 (1.275, 1.549)	1.395 (1.266, 1.538)	1.393 (1.263, 1.537)
Q3	1499	1.720 (1.561, 1.896)	1.697 (1.540, 1.187)	1.641 (1.487, 1.811)
Q4	1809	1.994 (1.813, 2.192)	1.967 (1.789, 2.163)	1.886 (1.713, 2.077)
Q5	2214	2.656 (2.420, 2.916)	2.623 (2.389, 2.879)	2.475 (2.250, 2.722)
P for trend		< 0.001	< 0.001	< 0.001
Continuous (per SD)	7690	1.374 (1.339, 1.410)	1.370 (1.335, 1.406)	1.355 (1.318, 1.393)
Female				
Q1	1622	Reference	Reference	Reference
Q2	1829	1.330 (1.232, 1.436)	1.309 (1.212, 1.414)	1.304 (1.207, 1.408)
Q3	2207	1.560 (1.449, 1.681)	1.524 (1.414, 1.641)	1.508 (1.399, 1.626)
Q4	2498	1.676 (1.558, 1.802)	1.638 (1.523, 1.762)	1.608 (1.494, 1.731)
Q5	2910	2.083 (1.939, 2.237)	2.043 (1.902, 2.195)	1.992 (1.853, 2.142)
P for trend		< 0.001	< 0.001	< 0.001
Continuous (per SD)	11,066	1.283 (1.254, 1.311)	1.276 (1.248, 1.305)	1.261 (1.233, 1.289)

Abbreviations: *OR* odd ratio; *CI* confidential interval; *BMI* body mass index; *WC* waist circumference; *SBP* systolic blood pressure; *RHR* resting heart rate

Model 1: unadjusted

Model 2: adjusted for age

Model 3: adjusted for age, alcohol consumption, place of residence, smoking, physical exercise, SBP, RHR

**Fig. 1:**
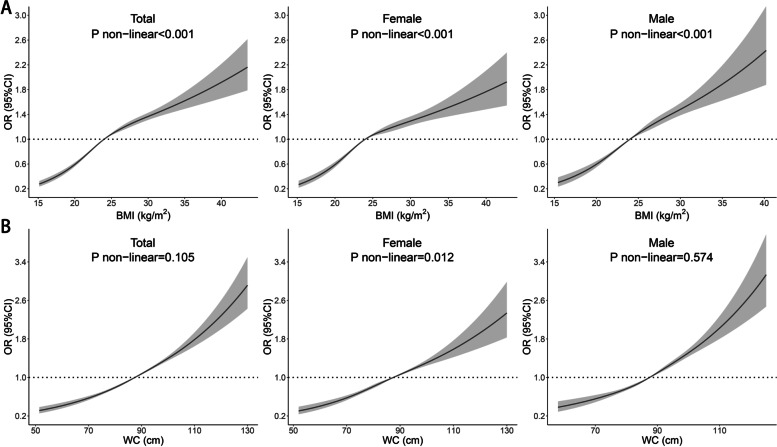
Relationship of BMI and WC with the risk of T2DM for all participants and subgroups of males and females. ORs are adjusted for age, sex (not for sex subgroup analysis), alcohol consumption, place of residence, smoking, physical exercise, SBP, RHR. Abbreviations: *BMI* body mass index; *CI* confidential interval; *OR* odd ratio; *WC* waist circumference; *SBP* systolic blood pressure; *RHR* resting heart rate

Multivariable adjusted restricted cubic spline analysis showed the dose–response relationship between BMI, WC and T2DM in Fig. 1, and the results showed that the risk of T2DM increased with increasing BMI and WC. The associations of BMI and T2DM were nonlinear in all participants and subgroups of sex, and the association of WC and T2DM was nonlinear in the female subgroup.

**Fig. 2:**
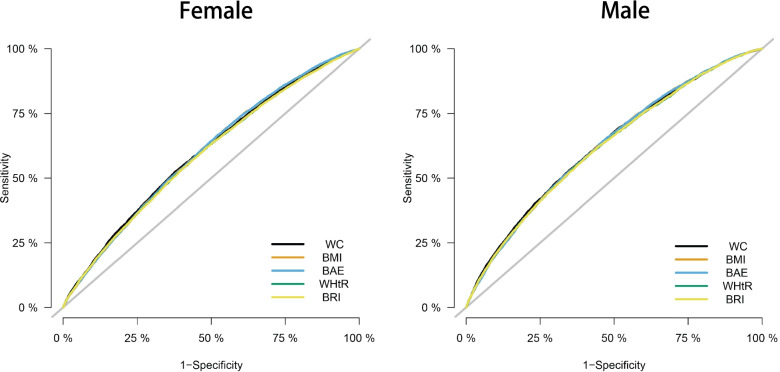
The receiver operating characteristic curve of anthropometric indicators after adjusting for age, sex, alcohol consumption, place of residence, smoking, physical exercise, SBP, RHR. Abbreviations: *BMI* body mass index; *WC* waist circumference; *WHtR* waist-to-height ratio; *BAE* body adiposity estimator; *BRI* body roundness index; *SBP* systolic blood pressure; *RHR* resting heart rate

The additive interaction of BMI and WC for T2DM was analyzed, and the results showed that the additive interaction did not exist (RERI: -0.059; 95% CI: -0.169 to 0.052; AP: -0.035; 95% CI: -0.100 to 0.030; S: 0.923; 95% CI: 0.797 to 1.068). As shown in Table 4, after adjusting for age, sex, place of residence, alcohol consumption, smoking, physical exercise, SBP, RHR, the AUCs of BMI, WC and other anthropometric indicators, including WHtR, BAE and BRI, for T2DM were calculated to compare the capability of those indices to identify T2DM. And the association of those anthropometric indicators with T2DM was shown in Supplementary Table 4. WHtR, BRI, BAE were positively associated with T2DM in both men and women. The ROCs are shown in Fig. 2. The best index to identify T2DM in males was WC (AUC: 0.629; 95% CI: 0.622 to 0.636), and in females, BMI (AUC: 0.600; 95% CI: 0.594 to 0.606), WC (AUC: 0.600; 95% CI: 0.593 to 0.606) and BAE (AUC: 0.600; 95% CI: 0.594 to 0.607) had a similar predictive ability for T2DM. When we adjusted for arbitrary combination of age, sex, place of residence, alcohol consumption, smoking, physical exercise, SBP and RHR (arbitrarily select 0–8 variables for adjustment), the results did not change significantly, so the findings were independent of the covariates considered.

**Table 4:** AUCs for anthropometric indices in relation to T2DM

Variable	AUC (95%CI)	Sensitivity	Specificity	Youden index
Male				
BMI (kg/m.^2^) + other factors	0.627 (0.620, 0.634)	0.641	0.539	0.180
WC (cm) + other factors	0.629 (0.622, 0.636)	0.532	0.651	0.183
WHtR + other factors	0.623 (0.616, 0.630)	0.555	0.622	0.177
BRI + other factors	0.623 (0.616, 0.630)	0.582	0.594	0.176
BAE + other factors	0.627 (0.620, 0.634)	0.642	0.539	0.181
Female				
BMI (kg/m.^2^) + other factors	0.600 (0.594, 0.606)	0.612	0.533	0.145
WC (cm) + other factors	0.600 (0.593, 0.606)	0.523	0.623	0.146
WHtR + other factors	0.593 (0.586, 0.599)	0.625	0.510	0.135
BRI + other factors	0.592 (0.586, 0.599)	0.618	0.517	0.135
BAE + other factors	0.600 (0.594, 0.607)	0.663	0.482	0.145

Abbreviations: *AUC* area under the curve; *BMI* body mass index; *BAE* body adiposity estimator; *BRI* body roundness index; *WC* waist circumference; *WHtR* waist-to-height ratio; *T2DM* type 2 diabetes mellitus; *SBP* systolic blood pressure; *RHR* resting heart rate

Other factors: age, sex, alcohol consumption, place of residence, smoking, physical exercise, SBP, RHR

## Discussion

Logistic regression was used in our study to examine the relationship between obesity and T2DM in the elderly, and increased BMI and WC were found to be associated with an increased risk of T2DM. The same results were found in the sex subgroup analysis. The dose–response relationship analyzed by restricted cubic splines found a nonlinear relationship between BMI and T2DM, while the relationship between WC and T2DM was nonlinear only in women. There was no additive interaction between BMI and WC for T2DM. Both BMI and WC were positively associated with T2DM. Meanwhile, we found that WC was the best predictor in elderly males, while BMI, WC and BAE had similar predictive abilities in females. When models were adjusted for arbitrary 0–8 variables of age, sex, place of residence, alcohol consumption, smoking, physical exercise, SBP and RHR, the results did not change significantly and the effect of potential confounding factors was eliminated, so the findings were independent of the covariates considered, in other words, the relationships of these anthropometric indicators with diabetes always exist no matter what covariates were adjusted in models.

Our study found that BMI and WC were positively associated with T2DM, and those positive associations were also found in some other studies [9, 24, 25]. There are several explanations for this positive association. First, people with genetic susceptibility to T2DM have a higher risk of obesity because the skeletal muscle and pancreas islet α-cells of those people are more prone to insulin resistance, and this insulin resistance leads to increased glucose production of the liver, raising insulin levels, which leads to obesity [26]. Second, macrophages in adipose tissue produce proinflammatory cytokines that influence insulin-dependent tissues and beta cells [27]. Third, the adipokine hypothesis [28] suggests that stressed adipokines release various secretory products that can affect insulin insensitivity and beta cells. Fourth, Martin G Myers Jr et al. [29] suggested that a high energy and fat diet can lead to dysfunction of the mitochondria and endoplasmic reticulum of the hypothalamus, resulting in leptin and insulin resistance. Increased leptin leads to the release of multiple inflammatory factors. Several studies have shown that some treatments for obesity, such as lifestyle changes, drug interventions, and surgery, not only lead to weight loss but also improve type 2 diabetes [30–32]. This also suggested that obesity increases the risk of T2DM.

In this study, the positive relationship between WC and T2DM was stronger than that of BMI in males. In contrast, the association between BMI and T2DM was stronger in females. Similar results were found by Qiwei Ge et al. [10]. This situation may be due to differences in fat distribution between the sexes [33, 34]. The study of Xuefeng Ni et al. [35] showed that women over 50 years of age had significantly less visceral abdominal fat than men, while men had more muscle mass than women. This difference in fat distribution may result in BMI becoming a better indicator of the amount of fat in women, while WC is a better indicator in men. Hormonal differences between men and women may be one reason for the difference in fat distribution [36].

In this study, ROC curves and AUC were used to compare the predictive power of BMI, WC and other anthropometric indicators, including WHtR, BAE and BRI, for T2DM. WC was the strongest predictor of T2DM in men. This result is similar to several studies [9, 24, 37]. Qiwei Ge et al. [10] also found that WC was the strongest indicator to predict T2DM in elderly men. However, these studies were not identical to the indicators of our study. In women, BMI, BAE and WC had similar predictive power for T2DM. In contrast to our study, a cohort study in Japan [38] found that BRI was better than BMI and WC in predicting T2DM in both men and women. Ye Chang et al. (20) also found that BRI had better predictive ability than BMI and WC. This discrepancy may be because the age range of the participants was different. All participants in our study were older than 60 years. We noted that the AUCs in our study were relatively low even after adjusting for some potential confounding factors, which may be because our study subjects were elderly. Qiwei Ge et al. [10] looked at people aged 18–60 and over. The AUCs of all indicators in people over 60 years old were lower than those in groups between 18 and 60 years old.

There are some advantages of our study. First, the data of this study were obtained from a large-scale health check in Henan, China. Demographic and laboratory data were collected, and the sample size and statistical power were adequate. Second, height and weight in this study were objectively measured rather than self-reported, which can avoid discrepancies between participants' own reports and the actual situation. Third, the AUCs were used to compare the predictive ability of BMI and WC with other anthropometric indicators, including WHtR, BAE and BRI, for T2DM in elderly individuals, which is of practical value to improving related studies.

However, some limitations of this study should be noted. First, the subjects of this study were older than 60 years, so we could not compare the relationship between obesity and T2DM in other age groups or the predictive ability of these indicators for diabetes. Second, this study was a cross-sectional study, making it difficult to examine the causal relationship between exposure and outcome. Third, we adjusted for potential confounders, including age, sex, alcohol consumption, smoking, physical exercise, place of residence, SBP, RHR, but some potential factors may exist that we did not adjust for, and since we did not have hip circumference information, we could not study its predictive power.

## Conclusion

Overall, this study found that the increased BMI and WC were associated with an increased risk of T2DM. The same results were found in the sex subgroup analysis. There was no additive interaction between BMI and WC for T2DM. WHtR, BRI, BAE were positively associated with T2DM in both men and women. Meanwhile, we found that WC was the best predictor in older males, while BMI, WC and BAE had similar predictive abilities in females.

## Supplementary Information

**Additional file 1: Supplementary Figure 1.** Screening flowchart of participants. **Supplementary Table 1.** Baseline characteristics of the included participants according to different levels of WC. **Supplementary Table 2.** The Pearson correlations of all anthropometric indices. **Supplementary Table 3.** The effect size of anthropometric indices between groups. **Supplementary Table 4.** Associations between anthropometric measures and T2DM.

## Data Availability

The datasets generated and/or analyzed during the current study are not publicly available due to confidentiality requirements of third parties, but are available from the corresponding author on request.

## Abbreviations

BMI: Body Mass Index
WC: Waist Circumference
T2DM: Type 2 Diabetes Mellitus
WHtR: Waist-to-Height Ratio
BAE: Body Adiposity Estimator
BRI: Body Roundness Index
AUC: Area Under the receiver operating Characteristic Curve
OR: Odd Ratio
CI: Confidence Interval
CVD: Cardiovascular Disease
ROC: Receiver Operating Characteristic Curve
RHR: Resting Heart Rate
SBP: Systolic Blood Pressure
DBP: Diastolic Blood Pressure
SD: Standard Deviation
RERI: Relative Excess Risk due to Interaction
AP: Attributable Proportion due to interaction
S: Synergy index
