# Supplementary material for: Association of body mass index and waist circumference with type 2 diabetes mellitus in older adults: a cross-sectional study
Source: BMC Geriatr. 2022 Jun 7;22:489. doi: 10.1186/s12877-022-03145-w (PMC9175364; doi:10.1186/s12877-022-03145-w)
Supplement: Supplementary file 1 — Additional file 1: Supplementary Figure 1. Screening flowchart of participants. Supplementary Table 1. Baseline characteristics of the included participants according to different levels of WC. Supplementary Table 2. The Pearson correlations of all anthropometric indices. Supplementary Table 3. The effect size of anthropometric indices between groups. Supplementary Table 4. Associations between anthropometric measures and T2DM. [file 12877_2022_3145_MOESM1_ESM.docx]

**Supplementary Fig. 1** Screening flowchart of participants.

**Supplementary Table 1** Baseline characteristics of the included participants according to different levels of WC

| **Characteristics** |  |  | **WC, cm** |  |  |  |
| --- | --- | --- | --- | --- | --- | --- |
|  | **First quintile** | **Second quintile** | **Third quintile** | **Fourth quintile** | **Fifth quintile** | **P Value** |
| Male | WC < 80 | 80 ≤ WC < 85 | 85 ≤ WC < 89 | 89 ≤ WC < 95 | WC ≥ 95 |  |
| Female | WC < 79 | 79 ≤ WC < 83 | 83 ≤ WC < 87 | 87 ≤ WC < 93 | WC ≥ 93 |  |
| Number of participants | 12346 | 13857 | 13654 | 14731 | 14800 |  |
| Diabetes, % | 2364 (19.1) | 3255 (23.5) | 3706 (27.1) | 4307 (29.2) | 5124 (34.6) | <0.001 |
| Age, years | 72.3±7.5 | 71.5±7.1 | 71.0±6.9 | 71.1±6.6 | 71.1±6.4 | <0.001 |
| Female, % | 7491 (60.7) | 6805 (49.1) | 7325 (53.6) | 7892 (53.6) | 7966 (53.8) | <0.001 |
| BMI, kg/m^2^ | 21.5±2.5 | 23.4±2.3 | 24.8±2.4 | 26.0±2.5 | 28.6±3.1 | <0.001 |
| WC, cm | 74.1±4.2 | 81.3±1.5 | 85.4±1.5 | 90.2±1.9 | 99.6±5.3 | <0.001 |
| BRI | 2.95±0.63 | 3.65±0.51 | 4.17±0.55 | 4.77±0.64 | 6.06±1.12 | <0.001 |
| BAE | 30.90±7.07 | 31.64±6.95 | 33.76±6.95 | 35.18±6.94 | 37.93±7.08 | <0.001 |
| WHtR | 0.48±0.04 | 0.51±0.03 | 0.54±0.03 | 0.57±0.03 | 0.63±0.05 | <0.001 |
| Smoking, % |  |  |  |  |  | <0.001 |
| Never smokers | 11118 (90.1) | 12202 (88.1) | 12218 (89.5) | 13076 (88.8) | 13124 (88.7) |  |
| Former smokers | 126 (1.0) | 215 (1.6) | 223 (1.6) | 248 (1.7) | 271 (1.8) |  |
| Current smokers | 1102 (8.9) | 1440 (10.4) | 1213 (8.9) | 1407 (9.6) | 1405 (9.5) |  |
| Alcohol consumption, % |  |  |  |  |  | <0.001 |
| Never | 11881 (96.2) | 13200 (95.3) | 12977 (95.0) | 13871 (94.2) | 13777 (93.1) |  |
| Once in a while | 260 (2.1) | 393 (2.8) | 425 (3.1) | 486 (3.3) | 570 (3.9) |  |
| More than once a week | 62 (0.5) | 84 (0.6) | 87 (0.6) | 123 (0.8) | 163 (1.1) |  |
| Every day | 143 (1.2) | 180 (1.3) | 165 (1.2) | 251 (1.7) | 290 (2.0) |  |
| Physical exercise, % |  |  |  |  |  | <0.001 |
| Never | 8411 (68.1) | 9466 (68.3) | 8867 (64.9) | 9135 (62.0) | 9087 (61.4) |  |
| Once in a while | 254 (2.1) | 310 (2.2) | 367 (2.7) | 391 (2.7) | 408 (2.8) |  |
| More than once a week | 689 (5.6) | 891 (6.4) | 978 (7.2) | 947 (6.4) | 914 (6.2) |  |
| Every day | 2992 (24.2) | 3190 (23.0) | 3442 (25.2) | 4258 (28.9) | 4391 (29.7) |  |
| Rural areas, % | 1843 (14.9) | 1807 (13.0) | 2184 (16.0) | 2699 (18.3) | 2843 (19.2) | <0.001 |
| RHR, beat | 73.4±11.1 | 73.7±10.7 | 73.5±10.7 | 73.1±10.9 | 73.5±11.1 | <0.001 |
| SBP, mmHg | 137.3±19.0 | 138.7±18.3 | 140.5±18.2 | 143.3±18.9 | 145.2±19.0 | <0.001 |
| DBP, mmHg | 80.8±10.3 | 82.0±9.8 | 82.9±9.9 | 84.2±10.3 | 85.5±10.6 | <0.001 |

Abbreviations: *BMI* body mass index; *WC* waist circumference; *SBP* systolic blood pressure; *DBP* diastolic blood pressure; *SD* standard deviation; *RHR* resting heart rate.

**Supplementary Table 2** The Pearson correlations of all anthropometric indices

| Indices | BMI | WC | WHtR | BRI | BAE |
| --- | --- | --- | --- | --- | --- |
| BMI | 1 | 0.704* | 0.702* | 0.702* | 0.573* |
| WC | 0.704* | 1 | 0.867* | 0.861* | 0.286* |
| WHtR | 0.702* | 0.867* | 1 | 0.996* | 0.570* |
| BRI | 0.702* | 0.861* | 0.996* | 1 | 0.568* |
| BAE | 0.573* | 0.286* | 0.570* | 0.568* | 1 |

Abbreviations: *BMI* body mass index; *WC* waist circumference; *BMI* body mass index; *WC* waist circumference; *WHtR* waist-to-height ratio; *BAE* body adiposity estimator; *BRI* body roundness index.

*:P<0.001, statistically significant.

**Supplementary Table 3** The effect size of anthropometric indices between groups

| **Anthropometric indices** | **No diabetes (n=50,632)** | **Diabetes (n=18,756)** | ***P**** | **Cohen’s d (95% CI)** |
| --- | --- | --- | --- | --- |
| BMI, mean ± SD | 24.7±3.5 | 25.7±3.5 | <0.001 | 0.29 (0.28, 0.31) |
| WC, mean ± SD | 85.9±9.0 | 88.3±9.3 | <0.001 | 0.26 (0.25, 0.28) |
| WHtR, mean ± SD | 0.54±0.06 | 0.59±0.06 | <0.001 | 0.25 (0.23, 0.26) |
| BRI, mean ± SD | 4.30±1.26 | 4.61±1.32 | <0.001 | 0.24 (0.23, 0.26) |
| BAE, mean ± SD | 33.51±7.45 | 35.38±7.25 | <0.001 | 0.25 (0.38, 0.27) |

*: ANOVA for continuous variables was used to compare the difference between two groups.

**Supplementary Table 4** Associations between anthropometric measures and T2DM

|  | **WHtR** | **BRI** | **BAE** |
| --- | --- | --- | --- |
| **Male category** |  |  |  |
| Q1 | Reference | Reference | Reference |
| Q2 | 1.318 (1.206, 1.441) | 1.329 (1.216, 1.454) | 1.319 (1.205, 1.443) |
| Q3 | 1.508 (1.381, 1.646) | 1.516 (1.388, 1.656) | 1.614 (1.477, 1.764) |
| Q4 | 1.694 (1.552, 1.848) | 1.700 (1.558, 1.855) | 1.760 (1.612, 1.923) |
| Q5 | 2.114 (1.940, 2.304) | 2.125 (1.949, 2.317) | 2.201 (2.017, 2.401) |
| P for trend | <0.001 | <0.001 | <0.001 |
| Continuous (per SD) | 1.338 (1.299, 1.379) | 1.345 (1.304, 1.386) | 1.322 (1.286, 1.358) |
| **Female category** |  |  |  |
| Q1 | Reference | Reference | Reference |
| Q2 | 1.248 (1.159, 1.345) | 1.257 (1.167, 1.354) | 1.331 (1.235, 1.435) |
| Q3 | 1.439 (1.337, 1.548) | 1.449 (1.345, 1.560) | 1.521 (1.412, 1.639) |
| Q4 | 1.517 (1.410, 1.633) | 1.505 (1.399, 1.619) | 1.754 (1.629, 1.888) |
| Q5 | 1.777 (1.652, 1.912) | 1.779 (1.653, 1.914) | 1.996 (1.855, 2.148) |
| P for trend | <0.001 | <0.001 | <0.001 |
| Continuous (per SD) | 1.208 (1.182, 1.235) | 1.196 (1.171, 1.221) | 1.271 (1.243 1.301) |

Abbreviations: *OR* odd ratio; *CI* confidential interval; *BMI* body mass index; *WC* waist circumference; *BMI* body mass index; *WC* waist circumference; *WHtR* waist-to-height ratio; *BAE* body adiposity estimator; *BRI* body roundness index; *SBP* systolic blood pressure; *RHR* resting heart rate.

Models adjusted for age, sex, alcohol consumption, smoking, physical exercise, place of residence, SBP, RHR.
